# Prime-Boost Immunizations with DNA, Modified Vaccinia Virus Ankara, and Protein-Based Vaccines Elicit Robust HIV-1 Tier 2 Neutralizing Antibodies against the CAP256 Superinfecting Virus

**DOI:** 10.1128/JVI.02155-18

**Published:** 2019-04-03

**Authors:** Michiel T. van Diepen, Rosamund Chapman, Nicola Douglass, Shireen Galant, Penny L. Moore, Emmanuel Margolin, Phindile Ximba, Lynn Morris, Edward P. Rybicki, Anna-Lise Williamson

**Affiliations:** a Institute of Infectious Disease and Molecular Medicine, Faculty of Health Science, University of Cape Town, Cape Town, South Africa; b Division of Medical Virology, Department of Pathology, University of Cape Town, Cape Town, South Africa; c Centre for HIV and STIs, National Institute for Communicable Diseases of the National Health Laboratory Service, Johannesburg, South Africa; d Faculty of Health Sciences, University of the Witwatersrand, Johannesburg, South Africa; e Centre for the AIDS Programme of Research in South Africa (CAPRISA), University of KwaZulu-Natal, Congella, South Africa; f Biopharming Research Unit, Department of Molecular and Cell Biology, University of Cape Town, Cape Town, South Africa; Icahn School of Medicine at Mount Sinai

**Keywords:** CAP256 SU, DNA, MVA, tier 2 neutralizing antibodies, human immunodeficiency virus, prime-boost, vaccine

## Abstract

A vaccine regimen that elicits broadly neutralizing antibodies (bNAbs) is a major goal in HIV-1 vaccine research. In this study, we assessed the immunogenicity of the CAP256 superinfecting viral envelope (CAP256 SU) protein delivered by modified vaccinia virus Ankara (MVA) and DNA vaccines in different prime-boost combinations followed by a soluble protein (P) boost. The envelope protein (Env) contained a flexible glycine linker and I559P mutation. Trimer-specific bNAbs PGT145, PG16, and CAP256 VRC26_08 efficiently bound to the membrane-bound CAP256 envelope expressed on the surface of cells transfected or infected with the DNA and MVA vaccines. The vaccines were tested in two different vaccination regimens in rabbits. Both regimens elicited autologous tier 2 neutralizing antibodies (NAbs) and high-titer binding antibodies to the matching CAP256 Env and CAP256 V1V2 loop scaffold. The immunogenicity of DNA and MVA vaccines expressing membrane-bound Env alone was compared to that of Env stabilized in a more native-like conformation on the surface of Gag virus-like particles (VLPs). The inclusion of Gag in the DNA and MVA vaccines resulted in earlier development of tier 2 NAbs for both vaccination regimens. In addition, a higher proportion of the rabbits primed with DNA and MVA vaccines that included Gag developed tier 2 NAbs than did those primed with vaccine expressing Env alone. Previously, these DNA and MVA vaccines expressing subtype C mosaic HIV-1 Gag were shown to elicit strong T cell responses in mice. Here we show that when the CAP256 SU envelope protein is included, these vaccines elicit autologous tier 2 NAbs.

**IMPORTANCE** A vaccine is urgently needed to combat HIV-1, particularly in sub-Saharan Africa, which remains disproportionately affected by the AIDS pandemic and accounts for the majority of new infections and AIDS-related deaths. In this study, two different vaccination regimens were compared. Rabbits that received two DNA primes followed by two modified vaccinia virus Ankara (MVA) and two protein inoculations developed better immune responses than those that received two MVA and three protein inoculations. In addition, DNA and MVA vaccines that expressed mosaic Gag VLPs presenting a stabilized Env antigen elicited better responses than Env alone, which supports the inclusion of Gag VLPs in an HIV-1 vaccine.

## Abstract (precis)

A vaccine is urgently needed to combat HIV-1, particularly in sub-Saharan Africa, which remains disproportionately affected by the AIDS pandemic and accounts for the majority of new infections and AIDS-related deaths. In this study, two different vaccination regimens were compared. Rabbits that received two DNA primes followed by two modified vaccinia virus Ankara (MVA) and two protein inoculations developed better immune responses than those that received two MVA and three protein inoculations. In addition, DNA and MVA vaccines that expressed mosaic Gag VLPs presenting a stabilized Env antigen elicited better responses than Env alone, which supports the inclusion of Gag VLPs in an HIV-1 vaccine.

## INTRODUCTION

Given the magnitude and duration of the human immunodeficiency virus (HIV)/AIDS pandemic, prophylactic vaccines against HIV type 1 (HIV-1) are urgently required. However, any prophylactic vaccine against HIV-1 will need to contend with the unprecedented diversity of the virus (1). While most successful antiviral vaccines confer protective immunity by the elicitation of neutralizing antibodies, this has been difficult to achieve for HIV due to several protective mechanisms inherent in the structure of the viral envelope (Env) glycoprotein. These include low spike density of the glycoprotein on the virion, poor accessibility of vulnerable epitopes, a host-derived glycan shield, and the presence of aberrantly folded glycoprotein species which misdirect the immune response against nonneutralizing epitopes, among other features (2–7).

Encouragingly, during natural infection approximately 30% of infected people develop broadly neutralizing antibodies (bNAbs) that can neutralize diverse viral isolates from different clades (8–16). Although these responses arise too late in infection to be of any obvious clinical benefit, their ability to protect against challenge with chimeric simian-human immunodeficiency virus (SHIV) in nonhuman primate (NHP) models suggests that they could protect against infection in humans (8, 14, 17–26). While the NAbs target a continuum of epitopes on the glycoprotein, many of these preferentially, or in some cases exclusively, recognize trimeric Env, suggesting that a vaccine immunogen should mimic this feature of the protein (27, 28).

HIV-1 isolates are ranked based on their sensitivities to neutralizing antibodies as tier 1 (sensitive), tier 2 (moderate), and tier 3 (resistant) (29). Tier 1 viruses are rarely isolated from natural infection and exhibit unusually high levels of conformational plasticity that exposes neutralization-susceptible epitopes (30, 31). Tier 2 isolates are representative of circulating viruses that a vaccine will need to confer protection against (29). The first generation of HIV-1 Env trimers was poorly representative of the native glycoprotein complex and elicited poor neutralizing antibodies against tier 2 viruses (29–31). This was partly due to the incorporation of heterologous trimerization motifs and the elimination of the native cleavage site to prevent shedding of the gp120 and gp41 subunits following proteolytic cleavage (32–39). It was subsequently reported that elimination of the Env cleavage site compromised the structure of the trimer, resulting in irregular conformations that exposed epitopes that are occluded in the native trimer (40, 41).

The importance of proteolytic cleavage in Env trimer formation was addressed by mutating the cleavage site to a hexa-arginine motif, which improved cleavage efficiency upon cotransfection with a second plasmid expressing furin (42). The sequence was then modified to introduce an artificial disulfide bond (SOS) between the gp41 ectodomain and gp120 to prevent shedding of gp120 and by the incorporation of an isoleucine-to-proline (I→P) helix-breaking mutation in gp41 (43, 44). The design was further refined to prevent aggregation by the elimination of a portion of the membrane-proximal external region (45). The incorporation of these “SOSIP” mutations into the prototype subtype A isolate, BG505, resulted in the first Env mimetic that accurately reproduced the native trimer structure (46). This approach has now been applied to a number of different Env proteins, resulting in the induction of strain-specific tier 2 virus-neutralizing antibodies in both rabbits and macaques (47–52).

A potential shortcoming of this approach is the requirement for furin coexpression, which limits its utility for delivery by genetic immunization or other vector modalities (42). In the absence of furin coexpression, the efficiency of cleavage is typically low, although this is influenced by the genetic background of the viral Env (53). To illustrate this point, *in vitro* expression of the prototypic BG505 Env from recombinant chimpanzee adenovirus and modified vaccinia virus (VACV) Ankara (MVA) vaccines only resulted in 66% and 33% respectively, of the recombinant Env being in the cleaved, native-like conformation (54).

A strategy employing a flexible glycine-rich linker peptide at the interface of the gp120 and gp41 subunits has been reported to enable recombinant Env to assume a native conformation in the absence of proteolytic cleavage (55). Immunization of animals with these Env trimers results in the induction of neutralizing antibodies that are comparable to those elicited by SOSIP antigens (51, 52). These Env mimetics are more suitable than the SOSIP constructs for heterologous prime-boost immunizations where endogenous furin is limited and coexpression is not possible *in vivo*.

A further iteration of Env trimer-based vaccination is the multivalent display of the glycoprotein trimer on the surface of liposomes or self-assembling nanoparticles (56–60). A more natural way to achieve this is to present the envelope glycoprotein on the surface of Pr55Gag virus-like particles (VLPs). Virus-like particle vaccines have been shown to be highly immunogenic and to provide protection from a number of viral diseases, such as hepatitis B virus and human papillomavirus infections (61, 62). Due to the repetitive nature of the envelope protein on the surfaces of VLPs, liposomes, and nanoparticles, cross-linking of B cell receptors specific to Env is triggered leading to improved antibody responses. These approaches are also thought to stabilize the trimeric conformation of the glycoprotein in its natural membrane context (63).

Gag is also believed to be an important component of an HIV vaccine, given that Gag-specific CD4^+^ and CD8^+^ T cells are inversely correlated with viral loads during natural infection (64–66). While bNAbs against the envelope glycoprotein are expected to prevent infection, cellular immunity against Gag could play a role in controlling viremia and possibly even ameliorate pathogenesis or reduce transmission of the virus (67). In addition to the elicitation of bNAbs, several other approaches have also been suggested to contend with the global diversity of the virus, such as the elicitation of cellular immunity against conserved regions of the HIV genome and the use of mosaic immunogens (1).

These mosaic antigens are designed *in silico* for the maximum coverage of potential T cell epitopes from a given number of natural sequences (68). Encouragingly, HIV mosaic vaccines have been reported to elicit cell-mediated immunity against HIV with improved breadth and confer protection against stringent SHIV challenges in nonhuman primates (69, 70). We have previously reported the formation of enveloped VLPs budding from cells transfected with DNA or infected with recombinant modified vaccinia virus Ankara encoding an HIV-1 subtype C Gag mosaic antigen (71). This antigen was also reported to be considerably more immunogenic than a comparable naturally occurring Gag and has demonstrated promising immunogenicity in DNA, MVA, and Mycobacterium bovis BCG vaccine modalities in mice (71, 72). A heterologous DNA prime-MVA boost regimen generated significantly improved T cell responses compared to homologous vaccination with either the DNA or MVA vaccine. Other groups have also shown that heterologous prime-boost regimens give potent HIV-1-specific immune responses that are often better than those generated with homologous vaccination regimens (73–75).

In this study, we have combined several of the most promising approaches reported in recent years to develop an optimal vaccine regimen for the elicitation of neutralizing antibodies to HIV-1 subtype C Env in a rabbit model. These include rational selection of an HIV-1 isolate for vaccine design, priming immune responses sequentially with DNA and MVA vaccines that express mosaic Gag VLPs presenting a stabilized Env antigen, and the use of a stabilized size exclusion chromatography (SEC)-purified protein as a boost.

## RESULTS

### *In vitro* characterization of South African subtype C CAP256 Env and mosaic Gag vaccines.

**FIG 1:**
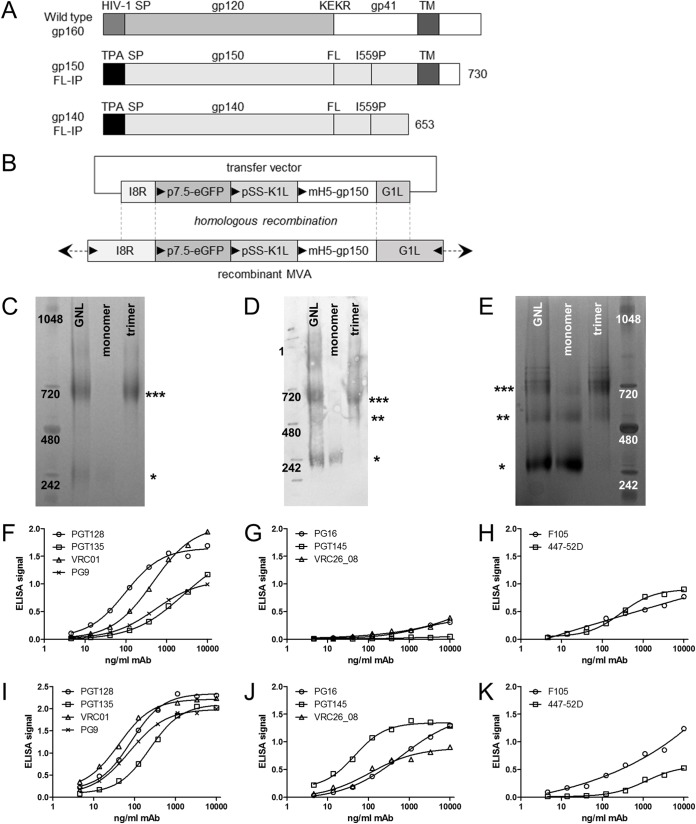
CAP256 DNA, rMVA and protein vaccine design and characterization of protein vaccine. (A) Schematic representation of wild-type Env and the truncated CAP256 gp150-FL-IP (gp150) used in DNA and MVA vaccines. The native signal peptide (HIV-1 SP) was replaced with the human tissue plasminogen activator (TPA SP) sequence, the furin cleavage site (KEKR) was replaced with a flexible linker (FL) sequence, and an I559P mutation was introduced. This sequence was further truncated for soluble gp140-FL-IP (soluble Env) protein vaccines. (B) Schematic representation of transfer vector for targeting gp150, expressed by the mH5 promoter, into the I8R-G1L locus of wild-type MVA or rMVA Gag^M^. Triangles indicate direction of open reading frames. (C and D) Soluble Env was purified from a stable HEK293 cell line expressing CAP256 gp140-FL-IP by Galanthus nivalis lectin affinity chromatography followed by size exclusion chromatography. Coomassie staining (C) and anti-Env Western blotting (D) show purification of soluble, trimeric Env (***, trimer; **, dimer; *, monomer). (E) Coomassie staining of soluble Env-His purified from a stable HEK293 cell line expressing CAP256 gp140-FL-IP-6×His by Galanthus nivalis lectin affinity chromatography followed by size exclusion chromatography. (F to H) ELISA for binding of MAbs to soluble, trimeric Env-His (representative traces). (I to K) ELISA for binding of MAbs to His-tagged BG505 SOSIP.664 (representative traces). No ELISA signal was observed for ELISA control without protein (data not shown).

The envelope sequence used in this study was based on a virus isolated from a patient in the South African CAPRISA 002 acute infection cohort, patient CAP256, who developed bNAbs following a secondary infection of HIV-1 approximately 15 weeks after the primary infection (76). The CAP256 superinfecting viral envelope (CAP256 SU) was selected, as it elicited bNAbs in this donor (77) and is sensitive to several prototype broadly neutralizing monoclonal antibodies (40). In addition, the enhanced reactivity of this Env for certain bNAb precursors makes it an appealing candidate immunogen (78, 79). For DNA and recombinant MVA (rMVA) vaccines, the Env sequence was truncated to gp150 (amino acid 730) to increase envelope protein expression and stability of the recombinant *env* gene (79), while retaining the transmembrane domain and part of the intracellular C-terminal domain (schematic representation in Fig. 1A). The DNA and MVA subtype C mosaic Gag (DNA-Gag^M^ and MVA-Gag^M^) and soluble Env (gp140) protein vaccines were described previously (71, 72, 80).

Our expression vector pTHCapR, which contains a porcine circovirus enhancer sequence that gives increased antigen expression, was used as the vector backbone for the DNA vaccines (71, 81). To generate the recombinant MVA vaccines, gp150 was targeted between the transcriptionally convergent open reading frames of the essential I8R and G1L genes of either wild-type (WT) MVA or MVA-Gag^M^ (Fig. 1B) to generate MVA Env and MVA Env+Gag^M^, respectively. Selection of recombinant MVA, which expressed both the enhanced green fluorescent protein (eGFP) marker gene and the vaccinia virus K1L host range gene, was performed in RK13 cells (82, 83). High-titer rMVA stocks (>0.5 × 10^9^ PFU) were generated in RK13 cells, and correct gp150 insertion was confirmed by PCR and sequencing.

As reported previously (80), soluble Env isolated via Galanthus nivalis lectin (GNL) affinity chromatography was mainly in a trimeric conformation as measured by molecular weight on NativePAGE protein gels. This trimeric fraction was further purified by size exclusion chromatography (Coomassie blue [Fig. 1C] and anti-Env Western blotting [Fig. 1D]). More monomeric and dimeric Env was seen following lectin affinity purification of the His-tagged Env (Fig. 1E, GNL) than the untagged Env (Fig. 1C, GNL).

The quaternary structure of this soluble presumptively native trimeric Env was assessed by capturing His-tagged Env on nickel-coated enzyme-linked immunosorbent assay (ELISA) plates and assaying binding of human monoclonal antibodies (MAbs) to native/trimeric Env. For soluble trimeric Env, both the CD4 binding site (MAb VRC01) and the Env V3-glycan supersite of vulnerability (MAbs PGT128 and PGT135) were intact (*n* = 3; Fig. 1F shows representative traces). In addition, binding of V2-glycan MAb PG9 (which binds both monomeric and trimeric Env) was observed. MAbs PG16, PGT145, and CAP256 VRC26_08 are trimer-specific antibodies that bind the V2 apex and have been shown to neutralize cell entry of the CAP256 SU virus (84). Weak binding of PG16 and CAP256 VRC26_08 was detected (Fig. 1G). PGT145 failed to bind, but this could be due to the much lower affinity of this MAb for CAP256 SU (84). The ELISA was positive for 447-52D; this MAb binds the V3 loop of gp120, which is considered nonneutralizing and a measure for monomeric Env and badly folded trimeric Env, suggesting that there is impaired folding of soluble Env (Fig. 1H). A second nonneutralizing MAb, F105, which binds the CD4 binding site of monomeric Env, was also positive (Fig. 1H). The His-tagged Env control protein, BG505 SOSIP.664-His, had a similar binding profile for MAbs PGT128, VRC01, and F105 (Fig. 1I and K) but much better binding for the trimer-specific MAbs PG16, PGT145, and CAP256 VRC26_08 (*n* = 1 [Fig. 1J]). No background binding of MAbs in the ELISA setup was observed (*n* = 1 [data not shown]). In all, these data confirm that a portion of CAP256 soluble trimeric Env is in a native-like state.

**FIG 2:**
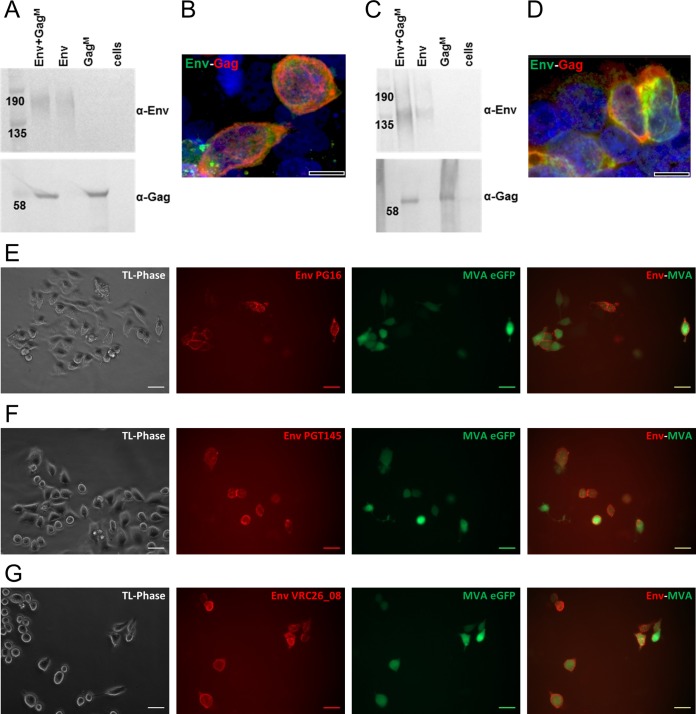
DNA and rMVA vaccine characterization. Western blots demonstrate *in vitro* secretion of Env and Gag protein from HEK293 cells after DNA vaccine transfection (A) or MVA vaccine infection (C). “Cells” refers to untransfected and uninfected cells. Membranes were cut in half and the top was probed with anti-Env antibodies and the bottom with anti-Gag antibodies. Confocal images show that both Env (green, Cy3) and Gag (red, Alexa Fluor 647) were expressed in the same cell when DNA vaccines were cotransfected (B) or when infected with rMVA containing both gp150 and Gag^M^ (D). Scale bars in confocal images represent 10 µm. (E to G) Live-cell staining of HeLa cells infected with MVA Env, using MAbs PG16 (E), PGT145 (F), and CAP256 VRC26_08 (G), which specifically detect native-like, trimeric Env. HeLa cells infected with rMVA were visualized by their eGFP expression (green). MAbs were detected with anti-human IgG-Cy3 (red). TL-Phase, transmitted light, phase contrast. Scale bars represent 20 µm.

Secreted Env and/or Gag expression was confirmed in media of HEK293T cells cotransfected with DNA Env+DNA Gag^M^, DNA Env, or DNA Gag^M^ vaccines, suggesting secretion of both proteins (Fig. 2A). Furthermore, both Gag and Env expression was observed within the same cells by confocal microscopy (Fig. 2B). Similar results were obtained for cells infected with the different rMVA vaccines (Fig. 2C and D).

The quaternary structure of Env expressed from the rMVA vaccines was further characterized by infecting HeLa cells with rMVA Env or rMVA Env+Gag^M^ and staining live cells using human MAbs visualized using an anti-human IgG-Cy3. Cells infected with rMVA Env or rMVA Env+Gag^M^ were identified by the expression of eGFP from rMVA. All the MAbs bound cells infected with rMVA Env to some degree (Table 1). Of note, binding by MAbs recognizing native-like trimers (PG16, PGT145, and CAP256 VRC26_08) (76, 84–87) was observed (Fig. 2E to G). Similar results were obtained for rMVA Env+Gag^M^ (Table 1) and DNA vaccines (Table 1). For DNA vaccines, expression of Env by transfected cells was identified using an anti-Env goat polyclonal antibody visualized with an anti-goat IgG-fluorescein isothiocyanate (FITC). Binding of the different MAbs to DNA and rMVA vaccines is summarized in Table 1. Untransfected and uninfected cells were negative in this assay (Fig. 2E to G and data not shown).

**TABLE 1:** Characterization of the Env expressed on the surface of cells infected with DNA and MVA vaccines^*a*^

Antibody	Neutralization	Epitope	Native-like trimer	Live-cell mapping		
				MVA Env	MVA Env+Gag^M^	DNV Env
PGT128	Broad	V3-glycan supersite	x	√	√	√
PGT135	Broad	V3-glycan supersite	x	√	√	√
447-52D	Narrow	V3	x	√	√	√
VRC01	Broad	CD4 binding site	x	√	√	√
F105	Narrow	CD4 binding site	x	√	√	√
PG9	Broad	V2 apex	x	√	√	√
PG16	Broad	V2 apex	Yes	√	√	√
PGT145	Broad	V2 apex	Yes	√	√	√
CAP256 VRC26_08	Broad	V2 apex	Yes	√	√	√
10E8	Broad	MPER	x	√	√	√

a Summary of Env MAbs which show positive binding in a live-cell staining assay to HeLa cells infected or transfected with MVA Env, MVA Env+Gag^M^, and DNA Env. √, positive for binding. x, antibody does not only bind Env in the native, trimeric state.

### *In vitro* virus-like particle formation of CAP256 Env and mosaic Gag vaccines.

**FIG 3:**
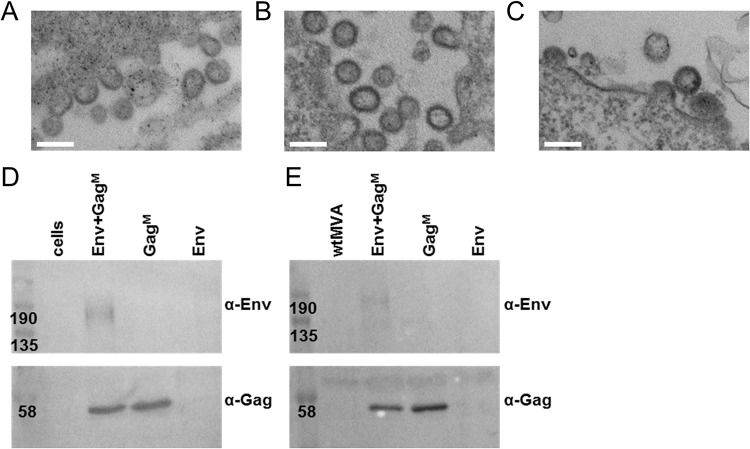
*In vitro* formation of virus-like particles (VLPs) from DNA and rMVA vaccines. VLPs, observed by transmission electron microscopy (TEM), after transfection of RK13 cells with DNA Gag^M^ (A) or cotransfection with DNA Env plus DNA Gag^M^ vaccines (B) are shown. (C) RK13 cells infected with rMVA Env+Gag^M^ at a multiplicity of infection (MOI) of 1 for 48 h. Scale bars represent 200 nm. VLPs from DNA (D) or rMVA (E) vaccines were isolated in a two-step OptiPrep gradient centrifugation protocol and analyzed by Western blotting. Fractions were isolated from untransfected cells (cells), cells transfected with DNA or infected with MVA vaccines expressing Env and Gag^M^ (Env+Gag^M^), Gag^M^ alone (Gag^M^), or Env alone (Env), and cells infected with wild-type MVA (wtMVA). (α-Env, anti-gp120; α-Gag, anti-p24). Membranes were cut in half and the top was probed with α-Env and the bottom with α-Gag.

Expression of the mosaic Gag from both DNA and MVA vaccines has already been shown to lead to the formation of Gag VLPs (71) and again was observed in this study (Fig. 3A to C). Inclusion of Env into these DNA or rMVA vaccines also resulted in VLP formation as assessed by electron microscopy (Fig. 3B and C). Gag VLPs were isolated in a two-step OptiPrep gradient centrifugation protocol for further characterization. For DNA Env+Gag^M^ and rMVA Env+Gag^M^ vaccines, both Gag and Env protein could be detected in the same fraction by Western blotting, suggesting that Env was associated with Gag in VLPs (Fig. 3D and E).

### Rabbit immunization and serum anti-Env antibody characterization.

**FIG 4:**
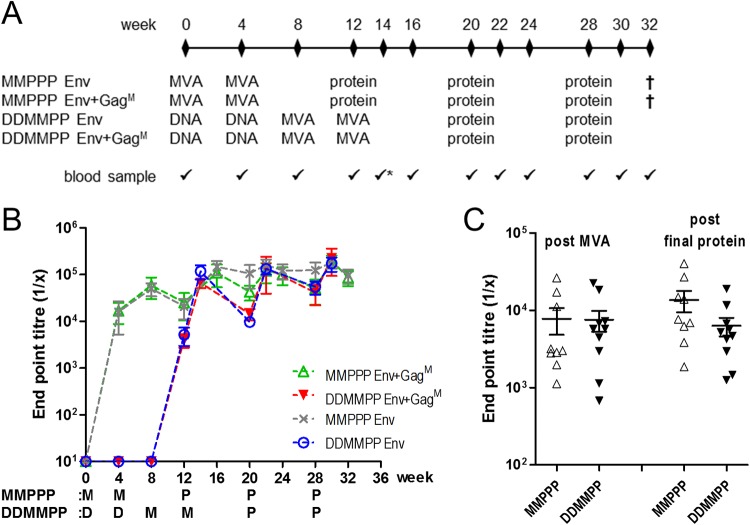
Rabbit immunization protocol and serum characterization. (A) Immunization regimen for the four different rabbit groups. DNA (D) and rMVA (M) vaccines expressed either gp150 and Gag^M^ or gp150 alone. DNA and MVA vaccines for DDMMPP were matched. All groups were boosted with soluble Env (P). (B) Time course of soluble CAP256 Env binding ELISA for rabbit sera. When no binding was observed, the endpoint titer was plotted as 10. (C) CAP256 V1V2 loop scaffold binding ELISA of rabbit sera after the second MVA inoculation (post MVA) or after the second protein boost (DDMMPP) or third protein boost (MMPPP) (post final protein). All data are presented as group averages ± SEMs.

The immunogenicity of the different vaccines was investigated by inoculating 4 groups of rabbits with different regimens (Fig. 4A). The first two groups received 10^8^ PFU of rMVA Env or rMVA Env+Gag^M^ intramuscularly at weeks 0 and 4, followed by three SEC-purified soluble, trimeric Env protein boosts (40 µg in AlhydroGel) at weeks 12, 20, and 28 (MMPPP). The other two groups received 100 µg of DNA Env or 100 µg of DNA Env plus 100 µg of DNA Gag^M^ plasmid at weeks 0 and 4, followed by two inoculations of 10^8^ PFU of rMVA Env or rMVA Env+Gag^M^ (matching the respective DNA primes) at weeks 8 and 12 and two protein boosts with 40 µg of SEC-purified soluble, trimeric Env protein at weeks 20 and 28 (DDMMPP). AlhydroGel was selected as an adjuvant because it was previously shown by us to elicit a superior immune response compared to that with unadjuvanted or the MF59-equivalent AddaVax adjuvanted GNL purified soluble Env protein (80).

Anti-Env antibody titers in rabbit sera for the different time points were measured in an autologous Env binding assay (Fig. 4B). Both Env+Gag^M^ and Env DNA vaccines failed to elicit anti-Env antibodies, which developed only after rMVA boosting. For both rMVA Env+Gag^M^ and rMVA Env, anti-Env antibodies were elicited after the first rMVA inoculation, and although protein boosting appeared to increase Env binding titers, there were no significant differences in the peak titers after MVA (week 8 for MMPPP or week 14 for DDMMPP) and protein (weeks 22 and 30) boosting. Env binding antibody titers were boosted after each inoculation and plateaued around a dilution of 1 × 10^−5^ for all 4 groups tested. Inclusion of Gag^M^ in the DNA or rMVA vaccines did not affect Env binding antibody titers in any of the regimens (two-way analysis of variance [ANOVA]).

Serum antibody binding against the autologous CAP256 SU WT V1V2 loop, which was presented on a scaffold to retain the natively folded state (88), was measured after the second MVA inoculation (week 6 for MMPPP and week 14 for DDMMPP) and 2 weeks after the final protein boost (week 30). The data from the two different groups in each regimen were combined (Fig. 4C). No significant differences in endpoint titers were observed between MMPPP and DDMMPP regimens or between post-MVA and post-final protein inoculations (two-way ANOVA). Similarly, when all groups were compared individually, inclusion of Gag^M^ in the DNA or rMVA vaccines did not affect Env V1V2 loop binding antibody titers (two-way ANOVA).

### Neutralization of HIV-1 pseudotype virions.

Rabbit sera were tested for neutralizing activity (reciprocal plasma/serum dilution causing a 50% reduction of relative light units [ID_50_]) against a panel of Env-pseudotyped viruses at selected time points (Table 2). In line with rMVA vaccines inducing anti-Env binding antibodies, neutralization titers for tier 1A virus MW965.26 and tier 1B virus 6644 were observed after the second rMVA inoculation (Table 2, MMPPP “primes” and DDMMPP “MVA B2”) in all groups. For MW965.26 these titers were significantly higher for the combined data of the two groups in the DDMMPP regimen than for the MMPPP groups (Student *t* test, *P* < 0.01). Protein boosting increased titers for MW965.26 in all four groups, and following the first protein boost, these titers were again significantly higher for the DDMMPP regimen (Student *t* test, *P* < 0.05). In line with this increased immunogenicity of the DDMMPP regimen, neutralizing antibodies against tier 1B 1107356 pseudovirions developed after MVA inoculation, whereas they developed only after protein boosting for the MMPPP regimen. Most encouragingly in these studies, all regimens induced vaccine-matched tier 2 (CAP256 SU) neutralization. For MMPPP groups, low-titer tier 2 neutralization was observed in the sera of 3/5 animals for MMPPP Env+Gag^M^ (ranging between 1:23 and 1:59) and 2/4 for MMPPP Env (1:28 and 1:333) after the third Env protein boost. These tier 2 neutralization titers were further enhanced by DNA priming, with 4/5 rabbits for DDMMPPP Env+Gag^M^ (ranging between 1:54 and 1:1,294) and 2/5 rabbits for DDMMPPP Env (1:74 and 1:204) developing these antibodies after the second Env protein boost. For 5/10 of the animals these titers were above the threshold required for 50% protection (1:105) in NHP models (89).

**TABLE 2:**
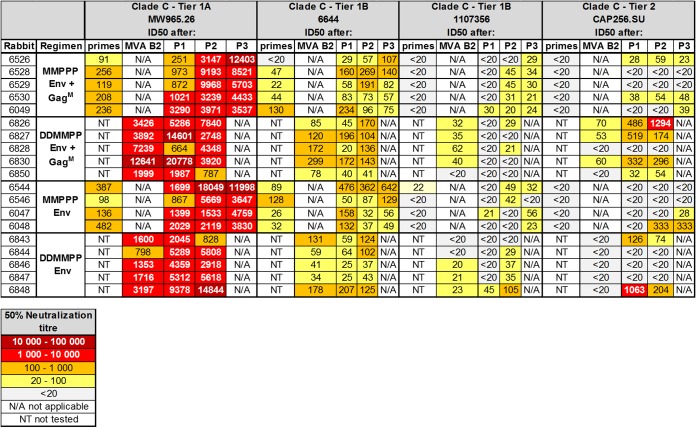
Serum neutralization measured by the TZM-bl assay^*a*^

a The serum tested was taken 4 weeks after the second MVA prime for MMPPPP (primes) or 2 weeks after the second MVA boost for DDMMPP (MVA B2) and 2 weeks after each protein inoculation (P1, P2, and P3). Neutralization titers of serum at week 0 were negative for all viruses tested. All serum from all time points were negative in the MuLV negative-control neutralization assay. The 50% neutralization titers are color-coded to reflect their potency range as indicated. Titers below 20 are considered nonneutralizing and not color-coded.

**FIG 5:**
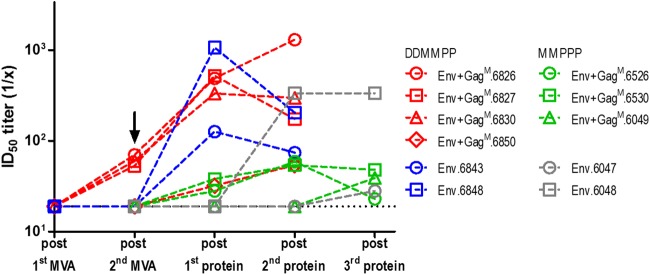
Serum neutralization measured by the TZM-bl assay. Longitudinal, tier 2 neutralizing antibody responses to autologous CAP256.SU pseudovirion from serum of individual rabbits are shown. The arrow indicates autologous tier 2 neutralization after the second rMVA boost in the DDMMPP regimen in rabbits receiving Env+Gag^M^ vaccines. For the MMPPP regimen, no neutralization assay was performed after the first rMVA vaccine. The dotted black line represents assay detection limit (1/20 dilution), all data points below the detection limit are plotted as 19.

Some interesting observations were made in a longitudinal analysis of the individual animals that developed autologous tier 2 neutralizing antibodies. In comparing the groups receiving Env+Gag^M^ with those receiving Env alone, it was observed that more animals developed autologous tier 2 neutralization when Gag was included in the vaccines, with 3 out of 5 for MMPPP Env+Gag^M^ (60%), compared to 2 out of 4 for MMPPP Env (50%); this was even more pronounced for DDMMPP regimens, with 4 out of 5 (80%) for Env+Gag^M^, compared to 2 out of 5 for Env alone (40%) (Fig. 5). However, these differences were not statistically different. Furthermore, in 5 out of the 7 animals that developed autologous tier 2 neutralizing antibodies, this occurred at an earlier time point when Gag was included in the vaccine, with only 1 animal having a delayed response. For the MMPPP regimen, autologous neutralization appeared after the first protein boost, whereas for the DDMMPP regimen, in 3 out of 4 rabbits, tier 2 neutralization of CAP256 SU developed after the second MVA inoculation, without the need for a protein boost (Fig. 5, arrow).

As autologous tier 2 neutralization was highest in animals treated with the DDMMPP regimen, week 30 sera from these particular rabbits were tested against a global panel of 10 tier 2 HIV-1 Env pseudoviruses. Three out of 6 animals developed a low titer neutralization response against clade A 398F1 (ranging between 1:20 and 1:29) (Table 3).

**TABLE 3:**
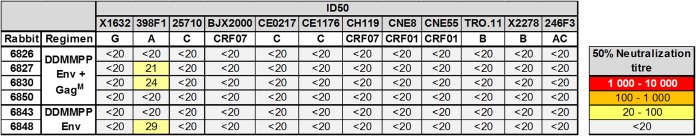
Serum neutralization of global tier 2 panel^*a*^

a The 50% neutralization titers are color-coded to reflect their potency range as indicated. Titers below 20 are considered nonneutralizing and not color-coded.

Serum from rabbits with autologous tier 2 neutralization titers at week 30 was used to investigate the possible site of neutralization within the Env sequence. A K169E mutation within CAP256 SU Env leads to the loss of binding of the native-like Env trimer-specific MAbs PG16 and CAP256 VRC26_08 (76, 77, 84). When sera were tested against CAP256 SU K169E pseudovirions, no differences were observed compared to CAP256 SU (Table 4). In line with this, chimeras formed by replacing the V1V2 region of two heterologous viruses, BG0505N332+ and CAP84, with that of CAP256 SU were not neutralized, indicating that the tier 2 NAbs elicited in this study probably did not target the V1V2 region.

**TABLE 4:**
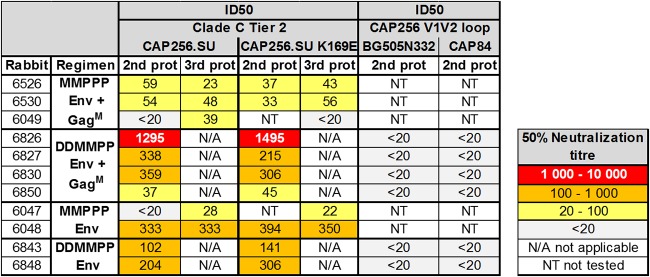
Characterization of serum neutralization epitope^*a*^

a Lack of neutralization of CAP256.SU K169E or neutralization of pseudovirions containing the V1V2 loop from CAP256 SU in heterologous CAP84 and BG505 backbones would reflect targeting of the trimer apex. The 50% neutralization titers are color-coded to reflect their potency range as indicated. Titers below 20 are considered nonneutralizing and not color-coded.

## DISCUSSION

An effective HIV-1 vaccine will need to induce a range of immune responses, including polyfunctional nonneutralizing antibody responses, broadly neutralizing antibody responses, and high frequencies of polyfunctional cytotoxic T cell responses to multiple epitopes (90–94). In a previous study, we showed that mice primed with DNA and boosted with MVA vaccines expressing HIV-1 subtype C mosaic Gag developed a strong, Gag-specific cellular immune response (71). The aim of the present study was to build on these results by developing an improved vaccine regimen for the elicitation of neutralizing antibodies against HIV-1 Env which could be tested in a rabbit model. The Env protein from the superinfecting CAP256 SU virus, which is thought to have elicited V1V2 bNAbs in CAPRISA donor 256, was selected as an immunogen (76, 77). A DNA vaccine vector with enhanced expression of transgenes due to a novel enhancer element from porcine circovirus type 1 was used to prime the immune response. This DNA vaccine has been demonstrated to markedly improve immunogenicity in mice and to allow significant dose sparing (71, 81). To the best of our knowledge, this is the first time DNA and MVA vaccines expressing a gp150 HIV-1 envelope protein containing a flexible glycine linker and I559P mutation have been utilized. Other groups have developed viral vaccines that express the soluble gp140 envelope protein containing a flexible linker (54), gp140 antigens with a mutated cleavage site and a trimerization domain at the C terminus (95), or gp150 and gp160 antigens with and without mutated cleavage sites (96–98). DNA and MVA vaccines expressing HIV-1 Env containing this flexible linker are more suitable than the SOSIP constructs for heterologous prime-boost immunizations where endogenous furin is limited and cleavage is inefficient, as no cleavage is required. Use of a flexible linker also ensures that the gp120 portion of the protein is not lost, leaving only gp41 stumps on the surface of VLPs. Lastly, inclusion of the transmembrane region and a portion of the cytoplasmic tail allows for the incorporation of the envelope protein into the cell membrane, which could also improve the stability and conformation of the Env protein.

The quaternary structure of CAP256 envelope expressed by cells transfected or infected with the DNA and MVA vaccines, respectively, was characterized by live-cell staining with a selection of bNAbs and MAbs. V2 binding site and trimer-specific bNAbs PGT145, PG16, and CAP256 VRC26_08 bound to CAP256 envelope expressed on the surface of the cells, as did bNAbs PGT128 and PGT135, which recognize the V3-glycan supersite. VRC01, which recognizes the CD4 binding site, also bound to cells expressing the modified CAP256 Env. The binding of these MAbs to our modified CAP256 Env suggests that a proportion of the Env protein is correctly folded in a native-like trimeric structure. The binding of F105, which recognizes disordered trimers, indicates that some nonnative-like envelope is also produced. This is not surprising, as Capucci et al. also reported a mixture of native-like and nonnative-like trimers expressed from simian adenovirus and MVA vectors (expressing the BG505 SOSIP.664 antigen) (54). Three out of four rabbits immunized with the simian adenovirus vaccine, followed by MVA and then ISCOMATRIX-adjuvanted BG505s trimer, developed autologous tier 2 NAbs. Similarly, in our study, four out of five rabbits vaccinated with the DDMMPP regimen developed autologous tier 2 NAbs to the CAP256 pseudovirus.

The three vaccines were tested in two different regimens (MMPPP and DDMMPP). Both regimens elicited autologous tier 2 neutralizing antibodies (Table 2) and high titers of binding antibodies to both the matching CAP256 Env and a CAP256 V1V2 loop scaffold (Fig. 4B and C). Neutralizing antibodies against HIV-1 have been shown to protect in nonhuman primates (NHPs) and are therefore seen as an important component of an HIV vaccine (99–101).

Higher mean peak titers of tier 2 NAbs were obtained for the DDMMPP regimen than for the MMPPP regimen, although these differences were not significant, probably due to the low numbers of animals used in the study. Some rabbits vaccinated with the DDMMPP regimen also developed low levels of neutralizing antibodies to clade A pseudovirus 398F1 (Table 3). These findings correlate with other studies which have shown that DNA primes a good humoral response. The addition of a DNA prime to MVA vaccination regimens also increases the magnitude and quality of T cell responses (102–104). Addition of DNA-C priming in the EV01 phase I trial increased IgG antibody responses against Env from 27% in the group vaccinated with NYVAC alone to 75% in the DNA+NYVAC group (102). The DNA prime also significantly boosted the T cell responses.

The inclusion of Gag in the DNA and MVA vaccines resulted in earlier development of tier 2 autologous neutralizing antibodies for both vaccination regimens. In addition, a higher proportion of the rabbits primed with vaccines that included Gag developed tier 2 autologous NAbs than those primed with DNA and MVA vaccines expressing Env alone. It is also interesting that three out of five rabbits developed low levels of autologous tier 2 NAbs after vaccination with the DNA and MVA vaccines when Gag was included in the vaccines, whereas rabbits vaccinated with DNA and MVA vaccines that did not express Gag (i.e., Env alone) developed tier 2 NAbs only after the first protein boost. This could be due simply to the improved adjuvant properties of a relatively large VLP, or it may be due to the stabilization of the Env in a more native-like conformation on the envelope of the Gag VLPs. It should be noted that the licensed vaccines against hepatitis B virus, rotavirus, and human papillomavirus are all VLPs, indicating that this is a highly effective mode of vaccine delivery (61, 62). Tong et al. (63) showed that the surface of HIV-1-derived VLPs often contains uncleaved gp160 and gp41 stumps that promote the development of nonneutralizing responses. The removal of these nonfunctional Envs with protease treatment resulted in VLPs that were better able to induce tier 2 NAbs (63, 96). As the Env used in this study contains a flexible linker between the gp120 and gp41, there is no necessity for cleavage, and thus, it is highly unlikely that there will be any exposed gp41 stumps on the surface of our VLPs. Ingale et al. (57) showed that SOSIP or NFL trimers presented on the surface of nanoparticles were better at activating germinal center B cells and inducing B cell receptor signaling and activation than soluble Env, further supporting the use of a particle-based HIV vaccine. The Env presented on the surface of these nanoparticles also showed a trend toward eliciting better neutralizing antibody responses than the soluble Env (57).

The structure of the His-tagged CAP256 gp140 protein was assessed by capture ELISA and detection with the same bNAbs and MAbs (Fig. 1). Again, both trimeric and nonnative species of Env were detected. Trimer-specific bNAbs PG16 and CAP256 VRC26_08 (Fig. 1G) recognized the CAP256 protein, but so did MAb F105 (Fig. 1H). In future studies, these nonnative trimers could be removed using negative selection with the F105 antibody. Additional mutations could also be introduced to improve the stability (105).

The CAP256 SU Env elicited potent V2-directed bNAbs during infection. Such antibodies typically recognize a lysine-rich region (residues 168 to 171) on Env and also interact with a glycan at N160 (78, 79, 88, 106). Furthermore, immunization of rabbits with a viral isolate, CRF250, that shares V2 apex sequence similarity with CAP256 SU (both contain a glycan hole at the V2 apex, which may enhance reactivity with V2 precursors) has recently been shown to elicit tier 2 autologous antibodies. Two of these NAbs were targeted to the lysine-rich basic region in the V2 apex, although these were shown not be to trimer specific, as they were adsorbed by gp120 proteins. In our study, we observed no neutralization of pseudoviruses containing V1V2 of CAP256 in heterologous CAP84 and BG505 backbones, and no effect was observed when point mutations were introduced into the C strand of the V2 region. This indicates that the tier 2 autologous NAbs elicited by this vaccine regimen targeted a region of the envelope other than V2. Further neutralization assays utilizing different chimeras and mutations will need to be carried out to determine the binding site(s) of the tier 2 NAbs elicited in this study.

The GOVX-B11 subtype B DNA prime-MVA boost vaccines, which have been tested in clinical trials and shown to be safe and immunogenic, are similar in design to the vaccines used in this study (97, 107). That DNA vaccine expresses Gag VLPs containing gp160 Env and granulocyte-macrophage colony-stimulating factor (GM-CSF), and the MVA vaccine expresses Gag VLPs containing gp150 Env. These envelopes do not contain modifications such as those used in the SOSIP or NFL trimers to improve native trimer formation and stability. The vaccines were found to elicit higher titers of antibodies to gp41 than gp120 and low levels of tier 2 neutralizing antibodies, in only 15% of vaccinees. The flexible linker and I559P mutation used in our vaccines should prevent the dissociation of the gp120 and gp41 subunits and better stabilize the Env trimer, consequently leading to improved antibody responses.

Although the rMVA used in this study contained the K1L vaccinia virus host range gene, which makes the virus replication competent in RK13 cells, it did not appear to result in replication in the rabbits, as evidenced by the monitoring of the weight and well-being of the animals. It is not known if the inclusion of K1L would affect immunogenicity of MVA.

Further studies are planned to optimize our vaccination regimen. These include (i) the use of Pharmajet, needle-free devices for inoculation of the DNA and, possibly, MVA vaccines, (ii) the use of different adjuvants, such as ISCOMATRIX, with the protein boosts, and (iii) subcutaneous rather than intramuscular inoculation. It would also be desirable to shorten the vaccination regimen to DMP as opposed to DDMMPP if a comparative immune response can be elicited. Such modifications may be amenable to sequential immunization strategies in which the priming antigen is followed by a series of immunogens to broaden the neutralizing antibody response (90, 91, 108).

The vaccines tested in this study have been shown to elicit high-titer binding antibodies to the V1V2 region of CAP256 Env and robust tier 2 neutralizing antibodies in rabbits. Previously, the DNA and MVA vaccines expressing HIV-1 Gag^M^ were shown to elicit strong T cell responses in mice (71). In addition, the induction of polyfunctional, nonneutralizing antibody responses should be assessed, as this type of response has been shown to play a critical role in protection in nonhuman primate challenge studies and to correlate with protection in the RV144 trial (90). The excellent responses demonstrated in this heterologous prime-boost regimen in a rabbit model justifies further testing of these novel candidate HIV-1 vaccines. Accordingly, plans are under way to test these vaccines in nonhuman primates.

## MATERIALS AND METHODS

### Antibodies, plasmids, cell lines, media, and reagents.

Goat anti-HIV-1 gp160 (MRC ADP 72 408/5104), rabbit anti-HIV-1 p24 (Gag) (ARP 432), donkey anti-goat IgG-Cy3 or -FITC, and donkey anti-rabbit IgG-Alexa Fluor 647 (Life Technologies) were used for immunofluorescence assays. Goat anti-human IgG (Fc specific)-Cy3 antibody (Sigma) was used for live-cell staining. Goat anti-HIV-1 gp120 (Bio-Rad; 5000-0557), goat anti-HIV-1 p24 (Gag) (Bio-Rad; 4999-9007), and mouse monoclonal anti-goat/sheep IgG-alkaline phosphatase (AP) GT34 (Sigma) were used for Western blotting. Anti-HIV-1 Env human monoclonal antibodies (MAbs) PG9, PG16, PGT128, PGT135, PGT145, CAP256 VRC26_08, VRC01, 10E8, F105, and 447-52D were expressed in FreeStyle 293F cells (Life Technologies) using the PEIMAX transfection reagent (Polysciences). Monoclonal antibodies were purified from cell-free supernatants after 6 days using protein A affinity chromatography (77). CAP256 SU V1V2 scaffolded proteins were purified by affinity chromatography using a nickel-nitrilotriacetic acid (Ni-NTA) column as described previously by Gorman et al. (78). Env control protein BG505 SOSIP.664-His trimers were purified by affinity chromatography using a Ni-Sepharose column as described previously (46).

HeLa, RK13, BHK-21, HEK293, and HEK293T cells were grown in Dulbecco’s modified Eagle’s medium (DMEM) (high glucose) plus l-glutamine (Lonza) plus 10% fetal calf serum plus 1× penicillin-streptomycin (Pen-Strep) (both from Gibco).

The mammalian expression plasmid pTHCapR was used as a backbone for all DNA vaccines (81), and pTJDNA4 containing mosaic *gag* (*gag*^M^) was described earlier (71). All DNA vaccines were synthesized by Aldevron.

### CAP256 gp150-FL-IP cloning.

The sequence of CAP256 SU gp160 (clone CAP256.206sp.032.C9) has been previously described (GenBank accession number KF241776.1) (84). This gene sequence was derived from that of the superinfecting virus isolated from patient CAP256 in the CAPRISA 002 cohort (76). The Env sequence was altered as follows and is schematically represented in Fig. 1A: the native leader (signal peptide [SP]) was removed and replaced with the human tissue plasminogen activator (TPA) leader sequence, the furin cleavage site was replaced with a flexible linker sequence (FL) (55), and an I548P mutation equivalent to the I559P in the SOSIP trimers was introduced to improve the trimerization of gp41 (44). Finally, the sequence was truncated to gp150 (amino acid [aa] 730) for MVA and DNA vaccines to increase expression and stability (109). Any potential poxvirus transcriptional termination signals (TTTTTNT) were removed from the coding sequence, and a poxvirus transcriptional termination sequence was added directly after the stop codon (TGA) of the Env gene. The Env sequences were human codon optimized and synthesized by GenScript. The DNA expression vector was called pMExT gp150-FL-IP (plasmid for mammalian expression with TPA leader). The TPA gp150-FL-IP was subcloned into the pox transfer vector.

### Recombinant MVA CAP256 Env constructs.

Wild-type modified vaccinia virus (VACV) Ankara (MVA) and recombinant MVA-Gag^M^ (71, 72) were used for targeted integration of CAP256 gp150-FL-IP. A transfer vector was created in which, in between flanking sequences of the MVA G1L-I8R locus, a selection cassette containing eGFP under the control of the VACV p7.5 promoter, K1L under the control of the VACV pSS promoter, and TPA-gp150-FL-IP under the control of the VACV mH5 promoter were inserted to form the plasmid Shuttle and Selection for Pox Expression (pSSPEx) CAP256 gp150-FL-IP. This plasmid allows for positive selection of integration into MVA via K1L selection in RK13 cells and identification of recombinant virus by eGFP expression.

BHK-21 cells were transfected with pSSPEx CAP256 gp150-FL-IP 2 h after infection with either wild-type MVA or rMVA-Gag^M^. Three days after infection and transfection, cells were freeze-thawed and the viral lysate was passaged in RK13 cells, a cell line permissive to MVA in the presence of K1L expression (82, 83). Single foci from RK13 cells were repeatedly selected and screened for correct integration of the targeting construct by PCR. Expression of Gag^M^ and/or Env was verified by Western blot analysis and immunofluorescence. Positive single foci were expanded in 3 hyperflasks and harvested after most RK13 cells had lifted. Three freeze-thaw cycles were performed, and supernatant containing rMVA virus was cleared by low-speed centrifugation. Remaining virus in the pellet was released by lysis with 0.1 mM Tris (pH 9), and the second supernatant was added to the previous one after another low-speed spin. rMVA was concentrated by centrifugation through a 36% sucrose cushion and resuspended in a total volume of 10 ml of PBS plus 10% glycerol. The generated rMVA Gag^M^ CAP256 gp150-FL-IP (rMVA Env+Gag^M^) and rMVA CAP256 gp150-FL-IP (rMVA Env) were aliquoted and stored at −80°C for downstream usage. Titers were determined in BHK-21 cells, in which GFP-positive plaques were counted 48 h after infection of a serial dilution range. Titers for both rMVA Env+Gag^M^ and rMVA Env were >0.5 × 10^9^ PFU/ml. High-titer stocks were screened for correct integration of the targeting construct by PCR and PCR sequencing (analyzed with CLC Main Workbench; Qiagen). Expression of mosaic Gag and/or Env was verified by Western blot analysis and immunofluorescence.

### Soluble CAP256 Env protein.

A stable cell line in HEK293 cells expressing CAP256 gp140-FL-IP protein (soluble Env) was made as described previously by van Diepen et al. (80). A second stable cell line was generated in a similar fashion for a His-tagged gp140 (soluble Env-His) in which a 3×Ala plus 6×His motif was added after amino acid 653 of CAP256 gp140-FL-IP. Soluble Env and soluble Env-His were isolated from media of respective serum-starved stable cell lines (passaged >10 times) as described previously. In short, flasks were coated with poly-l-lysine, allowing repeat harvests. Agarose-conjugated lectin (Galanthus nivalis; Sigma) (GNL) affinity purification columns were made to capture soluble Env from supernatant 3 days after changing to serum-free conditions. Protein was eluted in PBS plus 1 M methyl α-d-mannopyranoside (Sigma). Fractions containing trimeric protein (as assessed by molecular weight) were isolated after size exclusion chromatography (SEC) on a Superdex 200 HiLoad 16/600 column (GE Healthcare Life Sciences). Purified soluble trimeric Env protein was aliquoted and stored at −80°C for downstream usage. Protein concentration was determined using a DC protein assay (Bio-Rad) against a bovine serum albumin (BSA) standard.

### CAP256 GP140-FL-IP protein characterization.

To confirm isolation of soluble Env protein and trimers, precast NativePAGE Novex 3 to 12% bis-Tris protein gels (Life Technologies) were used for PAGE. Gels were either stained with Bio-Safe Coomassie (Bio-Rad) or blotted onto a polyvinylidene difluoride (PVDF) membrane (Bio-Rad). For the latter, blots were incubated with goat anti-gp120 (1:1,000), followed by monoclonal anti-goat/sheep IgG-AP (1:10,000), and detected with 5-bromo-4-chloro-3-indolylphosphate (BCIP)/nitroblue tetrazolium (NBT) phosphatase substrate (KPL). NativeMark unstained protein standard (Life Technologies) for native gel electrophoresis was used for estimating molecular weight. Liquid chromatography-mass spectrometry (CPGR, Cape Town) was used to confirm that soluble CAP256 Env was the foremost protein eluted off the lectin affinity purification column and in the trimeric fraction after SEC.

The trimeric fraction of soluble Env-His was used to characterize the antigenic structure. Ni-NTA 96 well plates (Qiagen) were coated with 200 ng/well of soluble, trimeric Env-His for 2 h, washed with PBS (3×), and blocked with 5% nonfat milk (Sigma) in PBS (block buffer) for 30 min (all at room temperature). Following PBS washing (3×), plates were incubated overnight at 4°C with 100 µl of serial dilutions (steps of 1:3) of anti-Env human monoclonal antibodies PG9, PG16, PGT128, PGT135, PGT145, CAP256 VRC26_08, VRC01, F105, and 447-52D in block buffer. Plates were washed with PBS (3×) and incubated with anti-human IgG HRP (1:10,000) (Dako) in block buffer. After the final PBS wash (3×), tetramethylbenzidene (TMB) ELISA substrate (Abcam) was added for detection and the reaction was stopped after 10 min with 100 µl of 1 N H_2_SO_4_. The signal was analyzed using a VersaMax ELISA microplate reader (Molecular Devices), which subtracted absorbance at 540 nm from 450 nm. The experiment was repeated for three different isolations (*n* = 3) and fitted to a four-parameter logistic regression curve (4PL curve) in GraphPad Prism 5.0.

### Expression and colocalization of mosaic Gag and CAP256 gp150-FL-IP.

To characterize Gag and Env expression and colocalization from rMVA and DNA vaccines, HEK293T cells were infected with MVA or transfected with DNA vaccines. After 48 h (rMVA vaccines) or 72 h (DNA vaccines), the supernatant was harvested and cleared by a low-speed spin (5 min at 275 × *g*) for Western blot analysis.

For immunofluorescence, cells were fixed with 4% paraformaldehyde for 10 min, permeabilized with methanol (1 min), and washed with PBS. Cells were blocked for 1 h at 37°C before being incubated with primary goat anti-gp160 and rabbit anti-p24 (both 1:500), diluted in blocking reagent, at 4°C overnight. After washing with PBS, secondary antibody (donkey anti-goat IgG-Cy3 and donkey anti-rabbit IgG Alexa Fluor 647, both at 1:500 in blocking reagent) was added for 1 h and coverslips were mounted with Mowiol after PBS washes. Cells were imaged on an LSM 880 Fast Airyscan (Zeiss) at the UCT Confocal & Light Microscope Imaging Facility. In figures, pseudocoloring for Cy3 is in red and that for Alexa Fluor 647 in green. Single images comprising 13 merged z-stacks were generated using Zeiss Zen software.

### Live-cell staining of CAP256 gp150-FL-IP using human monoclonal anti-Env antibodies.

To assess the structural integrity of Env expressed from rMVA and DNA vaccines, HeLa cells were infected with rMVA vaccines or transfected with DNA vaccines and tested for binding to anti-Env human monoclonal antibodies PG9, PG16, PGT128, PGT135, PGT145, CAP256 VRC26_08, VRC01, 10E8, F105, and 447-52D. After 48 h (rMVA vaccines) or 72 h (DNA vaccines) medium on cells was replaced with medium containing 10 µg/ml of MAb and incubated at room temperature for 1 h. For cells transfected with DNA vaccines, wells were incubated with goat anti-gp160 (1:500 in medium) at room temperature for 0.5 h. Following 3× PBS washes, cells were incubated with medium containing anti-human IgG-Cy3 (1:500) (for rMVA vaccines) or anti-human IgG Cy3 plus anti-goat IgG FITC (both 1:500) (for DNA vaccines) at room temperature for 0.5 h, and after 3× PBS washes, cells were imaged in PBS on a Zeiss Microscope using Zeiss Zen software.

### Detection of VLPs.

RK13 cells were infected with rMVA vaccines or transfected with DNA vaccines to investigate the formation of Gag virus-like particles (VLPs). After 48 h (rMVA vaccines) or 72 h (DNA vaccines) cells were collected by scraping, spun briefly, and washed, after which the pellets were washed with PBS and then were fixed overnight in 2.5% glutaraldehyde in PBS (4°C). The fixation agent was replaced with PBS, pellets were sent to the Microscopy & Microanalysis Unit at the University of KwaZulu-Natal for postprocessing as described earlier (71), and images were acquired on a JEOL 1010 platform.

HEK293T cells were infected with rMVA vaccines or transfected with DNA vaccines to characterize Env inclusion into VLPs. After 48 h (rMVA vaccines) or 72 h (DNA vaccines), VLPs were isolated from the supernatant of infected and transfected cells as follows. The supernatant was cleared with a low-speed spin (5 min at 275 × *g*) and run on an OptiPrep cushion (12, 24, and 60%) at ∼110,000 × *g* and 4°C for 90 min. A visible band (or equivalent) around the 12% to 24% interface was isolated and subsequently run overnight at ∼110,000 × *g* and 4°C on an OptiPrep gradient (18, 21, 24, 30, 36, and 42%). The only visible band (or equivalent) around 45 mm from the bottom was isolated. Bands from the cushion and gradient were analyzed for the presence of mosaic Gag or Env by Western blotting following separation on 8% denaturing SDS-PAGE gels. Western blotting was performed as described above, with both primary antibodies diluted at 1:1,000 and detected with AP-linked secondary antibodies (1:10,000).

### Rabbit immunization.

Female New Zealand White rabbits were housed in the animal facility of the Health Sciences Faculty at the University of Cape Town (UCT). All the animal procedures were approved by the UCT Animal Research Ethics Committee (reference UCT AEC 014-030 and 015-051) and performed by a trained animal technologist. Four groups, with 5 rabbits each, were selected to compare direct rMVA priming (rMVA Env+Gag^M^ or rMVA Env) with groups receiving DNA primes (DNA Env+Gag^M^ or DNA Env), followed by matching rMVA. All animals were boosted with trimeric, soluble CAP256 Env protein. DNA and rMVA priming vaccines were administered intramuscularly in the hind leg at weeks 0 and 4. DNA primes consisted of 100 µg of pTJDNA4 mixed with 100 µg of pMExT CAP256 gp150-FL-IP (both at 1 mg/ml) or 100 µg of pMExT CAP256 gp150-FL-IP (DDMMPP). Recombinant MVA was administered intramuscularly in the hind leg at weeks 0 and 4 as priming vaccines (MMPPP) or at weeks 8 and 12 as booster vaccines (DDMMPP) at 10^8^ PFU in 500 µl of PBS. For MMPPP groups, rabbits received 40 µg of trimeric soluble Env protein in 500 µl of 1:1 (vol/vol) Alhydrogel adjuvant in the same fashion at weeks 12, 20, and 28 to boost the immune response, whereas animals in the DDMMPP group received the protein boost at weeks 20 and 28. All animals were bled every 2 to 4 weeks from when inoculations started. Week 0 bleeds were used as prebleeds. In total, three animals died due to unrelated causes.

### Rabbit serum binding ELISAs.

To assess Env binding antibody titers in rabbit sera, a matching soluble CAP256 Env binding ELISA was performed as described earlier (80). In short, Nunc MaxiSorp flat-bottom 96-well plates (Sigma) were coated with 10 ng/well of soluble, trimeric Env. Rabbit sera were used in the primary incubation in a serial dilution range starting at 1:10. Anti-rabbit IgG-horseradish peroxidase (HRP) (1:5,000, Roche) was used for detection with TMB ELISA substrate (Abcam). The reaction was stopped after 10 min with 1 N H_2_SO_4_. The ELISA signal was analyzed using a VersaMax ELISA microplate reader (Molecular Devices), which subtracted absorbance values at 540 nm from values at 450 nm. ELISAs for the whole time course and each group were performed at the same time on duplicate plates. Duplicate data points were averaged and fitted to a four-parameter logistic regression curve (4PL curve) in GraphPad Prism 5.0. Antibody endpoint titers were calculated from 4PL curves with the threshold set as 4PL curve minimum + standard error of minimum for each time point. Data were plotted as the mean ± SEM for the whole group. ELISAs for binding to CAP256 SU V1V2 loop scaffold for week 0 and 2 weeks after the final MVA inoculation or protein boost were performed in a similar fashion, but wells were coated with 500 ng/well of protein.

### Rabbit serum neutralization assay.

Rabbit sera from different time points were tested for their ability to inhibit entry of Env-pseudotyped virions into a reporter cell line. Neutralization was measured as a reduction in luciferase gene expression after a single round of infection of JC53bl-13 cells, also known as TZM-bl cells (NIH AIDS Research and Reference Reagent Program), with Env-pseudotyped viruses (MW965.26, 6644, CA146, 1107356, CAP37, CT349, Du156, 188146, and CAP256 SU). Serum from selected animals and time points were also tested against CAP256 SU K169E, BG505 N332_CAP256 V1V2-loop, CAP84_CAP256 V1V2-loop and a Tier 2 global panel (X1632, 398F1, 25710, BJX2000, CE0217, CE1176, CH119, CNE8, CNE55, TRO.11, X2278, and 246F3). Titer was calculated as the reciprocal plasma/serum dilution causing a 50% reduction of relative light units (ID_50_). Dilutions were started at 1:20. For graphs, data were plotted as 19 when the ID_50_ was <20. Murine leukemia virus (MuLV) was used as a negative control.

### Statistical analysis.

All statistical analysis was performed in GraphPad Prism 5.0. Both one-way and two-way ANOVA were performed with Bonferroni *post hoc* testing. For the neutralization data, a titer value of 19 was used for data of <20.
